# High Levels of HIST1H2BK in Low-Grade Glioma Predicts Poor Prognosis: A Study Using CGGA and TCGA Data

**DOI:** 10.3389/fonc.2020.00627

**Published:** 2020-05-08

**Authors:** Weidong Liu, Zhentao Xu, Jie Zhou, Shuang Xing, Zhiqiang Li, Xu Gao, Shiyu Feng, Yilei Xiao

**Affiliations:** ^1^Department of Neurosurgery, Liaocheng People's Hospital, Liaocheng, China; ^2^Department of Nursing, Liaocheng Vocational and Technical College, Liaocheng, China; ^3^Department of Neurosurgery, Zhongnan Hospital of Wuhan University, Wuhan, China; ^4^Department of Neurosurgery, General Hospital of Northern Theater Command (General Hospital of Shenyang Military), Shenyang, China; ^5^Department of Neurosurgery, Chinese PLA General Hospital, Beijing, China

**Keywords:** HIST1H2BK, glioma, prognosis, CGGA, TCGA

## Abstract

A number of biomarkers have been identified for various cancers. However, biomarkers associated with glioma remain largely to be explored. In the current study, we investigated the relationship between the expression and prognostic value of the HIST1H2BK gene in glioma. Sequential data filtering (survival analysis, independent prognostic analysis, ROC curve analysis, and clinical correlation analysis) was performed, which resulted in identification of the association between the HIST1H2BK gene and glioma. Then, the HIST1H2BK gene was analyzed using bioinformatics (Kaplan–Meier survival analysis, univariate Cox analysis, multivariate Cox analysis, and ROC curve analysis). The results showed that low expression of HIST1H2BK was associated with better prognosis, and high expression of HIST1H2BK was associated with poor prognosis. In addition, HIST1H2BK was an independent prognostic indicator for patients with glioma. We also evaluated the association between HIST1H2BK and clinical characteristics. Furthermore, gene set enrichment analysis (GSEA) and analysis of immune infiltration were performed. The results showed that HIST1H2BK was associated with intensity of immune infiltration in glioma. Finally, co-expression analysis was performed. The results showed that HIST1H2BK was positively correlated with HIST1H2AG, HIST2H2AA4, HIST1H2BJ, HIST2H2BE, and HIST1H2AC, and negatively correlated with PDZD4, CRY2, GABBR1, rp5-1119a7.17, and KCNJ11. This study showed that upregulation of HIST1H2BK in low-grade glioma (LGG) tissue was an indicator of poor prognosis. Moreover, this study demonstrated that HIST1H2BK may be a promising biomarker for the treatment of LGG.

## Introduction

Glioma is one of the most common primary intracranial malignancies, and it encompasses two principle subgroups: diffuse gliomas and gliomas showing a more circumscribed growth pattern (“non-diffuse gliomas”) (1, 2). In the revised fourth edition of the WHO Classification of CNS tumors published in 2016, classification of gliomas was fundamentally changed: for the first time, a large subset of these tumors is now defined based on presence/absence of IDH mutation and 1p/19q codeletion (1, 3). Because the integrated histological-molecular classification was superior to a purely histological classification, WHO 2016 Classification of gliomas would be helpful for treatment (4, 5). However, the prognosis of glioma is still poor due to the infiltrative nature of this malignancy, and a high local relapse rate (6). Recent molecular advances have altered the field of neuro-oncology by allowing for identification of diagnostic and prognostic markers, and identification of therapeutic targets (7).

Advances in bioinformatics and high-throughput sequencing have resulted in identification of many tumor biomarkers that may aid the prognosis accuracy of glioblastoma multiforme (GBM), which may result in more effective management of this disease (8, 9). Circulating miR-128 was identified as a potential marker for early diagnosis of glioma (10). Increased expression of OPN is considered to be an indicator of poor prognosis of GBM (11). Furthermore, Zeng et al. suggested that TRPM8 may be a promising biomarker of GBM invasiveness, and a potential target for treatment of glioblastoma (12). In the future, these markers may be used for advanced diagnostic and decision-making processes. Despite these advances, more reliable prognostic indicators are needed for glioma (13).

In this present study, the HIST1H2BK gene was screened using data filtering (survival analysis, independent prognostic analysis, ROC curve analysis, and clinical correlation analysis). Then, bioinformatics analysis of HIST1H2BK was performed. In addition, the association between HIST1H2BK and clinical characteristics was investigated. Furthermore, gene set enrichment analysis (GSEA) and immune infiltration correlation analysis were performed. Finally, co-expression analysis was performed.

## Materials and Methods

### Data Download and Preprocessing

Gene expression data and corresponding clinical data from glioma patients were downloaded from CGGA (LGG+GBM) (http://www.cgga.org.cn/). Two datasets that contained 693 and 325 samples (DataSet ID: mRNAseq_693 and mRNAseq_325, Data Type: RNA sequencing) were downloaded. The two sets of gene expression data from glioma samples were corrected in batches and integrated by loading them into the limma (14) and sva (15) packages in R software (R version 3.6.1:https://www.r-project.org/).

### Survival Analysis Filtering

Survival and survminer packages were loaded in R software, and Kaplan–Meier (K-M) (16) and univariate Cox analyses were used to filter gene expression data and survival data at a significance level of *P* < 0.001.

### Gepia Database Analysis and HPA Database Analysis

There are two types of gliomas in the TCGA dataset, including LGG and GBM, corresponding to low-grade tumors and high-grade tumors, respectively. The examination of HIST1H2BK expression in homogeneous subsets of gliomas was performed in GEPIA. GEPIA, an interactive web server containing RNA sequencing data based on 9,736 tumor samples and 8,587 normal samples from the TCGA and GTEx databases, provides customizable functions such as tumor/normal differential expression analysis, patient survival analysis, and correlation analysis. To further assess the expression of HIST1H2BK from protein levels in normal tissues and tumor tissues, HPA (The Human Protein Atlas, http://www.proteinatlas.org) was also used to validate the immunohistochemistry of HIST1H2BK.

### Independent Prognostic Analysis Filtering

The gene expression data obtained from the survival analysis and integrated clinical information were analyzed using multivariate Cox analysis with R software, at a significance level of *P* < 0.001.

### Receiver Operating Characteristic Curve Analysis

The gene expression data obtained from independent prognostic analysis filtering were screened using survival ROC curve analysis (https://CRAN.R-project.org/package=survivalROC), at a criterion of AUC > 0.7.

### Clinical Relevance Filtering

The gene expression data obtained from ROC curve filtering and the corresponding clinical information were analyzed using R software and filtered using a threshold of *P* < 0.05.

### Bioinformatics Analysis

Gene expression data and corresponding clinical information obtained from ROC curve filtering were analyzed using R software, and HIST1H2BK gene expression data were analyzed with survival time, survival status, and other clinical traits. Survival and survminer tools were used to plot a survival curve for HIST1H2BK and glioma, and univariate Cox analysis and multivariate Cox analysis were performed. Considering the dependency of HIST1H2BK expression level on cell replication, the predictive value of HIST1H2BK was compared with that of Ki-67, a positive marker of the cell cycle. The survival ROC package was used in R software to generate ROC curves for HIST1H2BK, Ki-67 and glioma at 1, 3, and 5 years using the K-M method.

### Analysis of the Correlation Between HIST1HB2K Expression and Clinical Characteristics

Gene expression data and corresponding clinical information obtained from ROC curve filtering were analyzed in R to extract the clinical data associated with the HIST1H2BK gene. The correlation between HIST1H2BK expression and various clinical characteristics was plotted using beeswarm (https://CRAN.R-project.org/package=beeswarmpackage).

### Gene Set Enrichment Analysis

Gene set enrichment analysis is a computational method used to determine whether a group of genes is differentially expressed in two biological states (17). In this study, GSEA was used to generate an ordered list of all genes associated with the expression of HIST1H2BK. Then, GSEA was used to identify survival differences between the high and low HIST1H2BK groups. Gene set permutations were performed one thousand times for each analysis. The expression level of HIST1H2BK was used as a phenotype label. The false discovery rate (FDR) and normalized enrichment score (NES) were used to sort the gene ontology (GO) and KEGG pathways enriched in each phenotype.

### Analysis of Immune Infiltration

Tumor Immune Estimation Resource (TIMER, https://cistrome.shinyapps.io/timer/) was used to comprehensively study the molecular characteristics of tumor-immune interactions (18). This web server allows users to input function-specific parameters to generate dynamically displayed figures, which facilitates access to tumor immunological, clinical, and genomic features. The abundances of six immune infiltrates, including B cells, CD4+ T cells, CD8+ T cells, macrophages, neutrophils, and dendritic cells were evaluated. We analyzed the relationship between the expression level of HIST1H2BK and the level of immune infiltration in glioma using the TIMER “gene” module. The Kaplan-Meier method was used to plot the effect of HIST1H2BK expression and immune cell infiltration on the prognoses of patients with glioma (GBM+LGG), and clinical factors were included to construct a multivariate Cox proportional risk model. Finally, the relationship between copy number variations (CNVs) of HIST1H2BK in different somatic cells and the level of infiltration in glioma was analyzed using the SCNA module.

### Co-expression Analysis

The limma (14) package in R was used to screen genes that were co-expressed with HIST1H2BK. The thresholds for co-expression were a correlation coefficient >0.5 and *p* < 0.001. In addition, the pheatmap (https://github.com/taiyun/corrplot) package was used to plot the first 20 genes positively and negatively associated with HIST1H2BK. The Corrplot (https://github.com/taiyun/corrplot) and Circlize (19) packages were used to generate a circular plot of the top five genes positively and negatively associated with HIST1H2BK.

## Results

### Data Filtering

Survival analysis was performed using the K-M and univariate Cox methods, with a significance threshold of *P* < 0.001 (Supplementary Table 1). Multivariate Cox analysis was performed to filter the genes with *P* < 0.001 in the univariate Cox analysis (Supplementary Table 2). Area under the curve > 0.7 was set as the threshold for further ROC curve analysis of genes (Supplementary Table 3). Finally, the relationship between genes and clinical characteristics was analyzed, with *P* < 0.05 as the criterion for significance (Supplementary Table 4). These analyses resulted in selection of the HIST1H2BK gene for subsequent analysis.

### Bioinformatics Analysis of HIST1H2BK in CGGA Database

**Figure 1:**
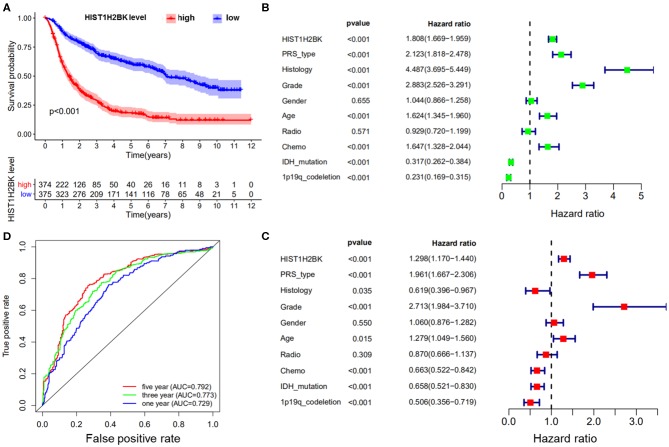
Bioinformatics analysis of HIST1H2BK using the CGGA database (LGG+GBM, *n* = 749). **(A)** Survival analysis of patients with glioma in the high HIST1H2BK and low HIST1H2BK groups. Red indicates high expression and blue indicates low expression. *P* < 0.001. **(B)** Univariate analysis of HIST1H2BK. **(C)** Multivariate analysis of HIST1H2BK. **(D)** Receiver operator characteristic curve analysis of HIST1H2BK. AUC, area under the curve.

Kaplan-Meier survival analysis of the CGGA dataset (including GBM and LGG) showed that low HIST1H2BK expression was associated with better prognosis in patients with glioma, and high expression of HIST1H2BK was associated with poor prognosis (Figure 1A). Univariate Cox analysis showed that HIST1H2BK (HR = 1.808; 95% CI = 1.669–1.959; *P* < 0.001), PRS type, histology, grade, age, and chemo were high-risk factors, and IDH mutation and 1p19qcodeletion were low-risk factors (Figure 1B). Multivariate Cox analysis showed that HIST1H2BK (HR = 1.298; 95% CI = 1.170–1.440; *P* < 0.001) was independently associated with overall survival, which suggested that HIST1H2BK could be an independent prognostic indicator for glioma. In addition, PRS type, grade, age, chemo, IDH mutation, and 1p19q codeletion may also be independent prognostic factors (Figure 1C). Receiver operating characteristic curve analysis showed that HIST1H2BK was a predictor of 1-year (AUC = 0.729), 3-year (AUC = 0.773), and 5-year survival (AUC = 0.792) (Figure 1D). The results of ROC analysis of Ki-67 showed poor predictive capability (1-year, AUC = 0.509; 3-year, AUC = 0.510; 5-year survival, AUC = 0.612). The predictive value of Ki-67 was lower than that of HIST1H2BK, which further demonstrated that HIST1H2BK was a good predictive marker.

### Relationship Between HIST1H2BK Expression and Prognosis of Glioma Patients

**Figure 2:**
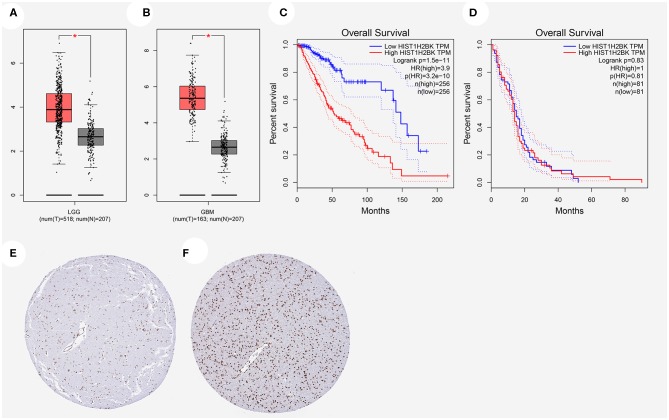
Relationship between HIST1H2BK expression and prognosis of glioma patients based on GEPIA database and HPA database. **(A)** HIST1H2BK was significantly upregulated in LGG. *P < 0.05. **(B)** HIST1H2BK was significantly upregulated in GBM. *P < 0.05. **(C)** The relationship between HIST1H2BK expression levels and overall survival in LGG analyzed by GEPIA database. **(D)** The relationship between HIST1H2BK expression levels and overall survival in GBM analyzed by GEPIA database. **(E)** Protein levels of HIST1H2BK in normal tissue by immunohistochemistry based on the Human Protein Atlas (staining: medium; intensity: moderate; quantity: >75%). **(F)** Protein levels of HIST1H2BK in LGG tissue by immunohistochemistry based on HPA (staining: high; intensity: strong; quantity: >75%). GEPIA, Gene Expression Profiling Interactive Analysis; HPA, the Human Protein Atlas; LGG, low grade glioma; GBM, glioblastoma; num, number; T, tumor; N, normal.

The expression of HIST1H2BK showed significant difference in LGG and GBM compared with that in normal control (Figures 2A,B). Prognostic analysis revealed that high expression of HIST1H2BK would lead to a short overall survival in patients with LGG (Figure 2C, *P* < 0.05), and the expression level of HIST1H2BK was not associated with the prognosis of patients with GBM (Figure 2D, *P* > 0.05). In addition, the protein level of HIST1H2BK was significantly higher in LGG tissues compared with normal tissues based on HPA (Figures 2E,F). The direct links to these images in HPA are as follows: https://www.proteinatlas.org/ENSG00000197903-HIST1H2BK/tissue/cerebral+cortex#img (HIST1H2BK in normal tissue); https://www.proteinatlas.org/ENSG00000197903-HIST1H2BK/pathology/glioma#img (HIST1H2BK in LGG tissue).

### Analysis of the Relationship Between HIST1H2BK Expression and Clinical Features

**Figure 3:**
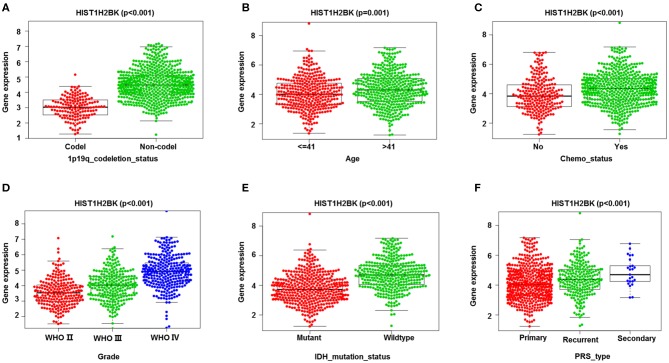
Correlation analysis between HIST1H2BK expression and clinical characteristics using the CGGA database (LGG+GBM, *n* = 749). Differential expression of HIST1H2BK was significantly related to **(A)** 1p19q codeletion status (Codel, *n* = 155; Non-codel, *n* = 594), **(B)** age (≤ 41, *n* = 342; >41, *n* = 407), **(C)** chemo status (No, *n* = 229; Yes, *n* = 520), **(D)** grade(WHO II, *n* = 218; WHO III, *n* = 240;WHO IV, *n* = 291), **(E)** IDH mutation status (Mutant, *n* = 410; Wildtype, *n* = 339), and **(F)** PRS type (Primary, *n* = 502; Recurrent, *n* = 222; Secondary, *n* = 25).

Analysis of 1,018 samples from the CGGA database **(including LGG and GBM)** showed that differential expression of HIST1H2BK was significantly associated with PRS type, histology, grade, age, chemo status, IDH mutation status, and 1p19q codeletion status (Figure 3, Supplementary Figure 1).

### Gene Set Enrichment Analysis of HIST1H2BK

**Figure 4:**
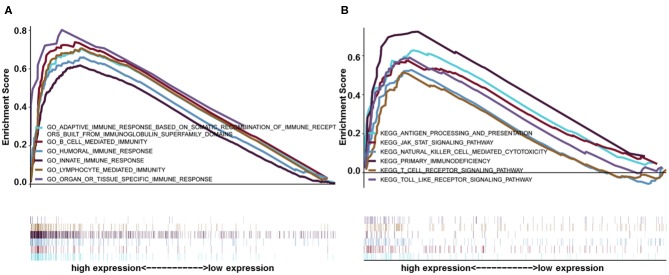
GSEA enrichment analysis of HIST1H2BK using the CGGA database (LGG+GBM, *n* = 749). **(A)** Gene ontology adaptive immune response based on somatic recombination of immune receptors built from immunoglobulin super family domains, B cell mediated immunity, humoral immune response, innate immune response, and lymphocyte mediated immunity. These GO terms were differentially expressed between the high and low HIST1H2BK phenotypes. **(B)** Antigen processing and presentation, JAK/STAT signaling pathway, natural killer cell mediated cytotoxicity, toll-like receptor signaling pathway, and primary immunodeficiency pathways were differentially enriched between the high and low HIST1H2BK phenotypes.

Gene set enrichment analysis was used to identify GO and signaling pathways that were differentially expressed in glioma (LGG+GBM) between the low and high HIST1H2BK expression groups. The results showed significant differences (FDR < 0.05) in enrichment using MSigDB Collection (c2.cp.biocarta and h.all. v6.1. symbols). The most significantly enriched GO and signaling pathways were selected based on a normalized enrichment score (NES). As shown in Figure 4, B cell related l mediated immunity, humoral immune response, innate immune response, lymphocyte mediated immunity Gene ontology terms (Supplementary Figure 2), antigen processing and presentation, the JAK-STAT signaling pathway, natural killer cell mediated cytotoxicity, toll-like receptor signaling pathway, primary immunodeficiency etc. pathways (Supplementary Figure 3) were enriched in the HIST1H2BK high expression phenotype.

### Analysis of the Correlation Between HIST1H2BK Expression and Clinical Characteristics in CGGA Database (LGG+GBM)

**Figure 5:**
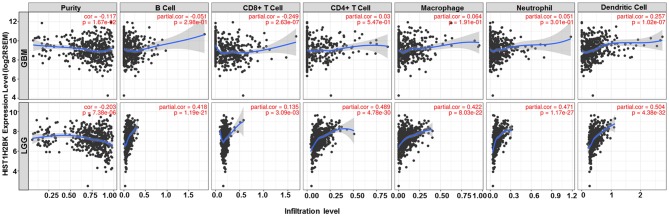
Correlation between the expression of HIST1H2BK and immune infiltration of glioma cells. GBM, glioblastoma; LGG, low grade glioma.

**Figure 6:**
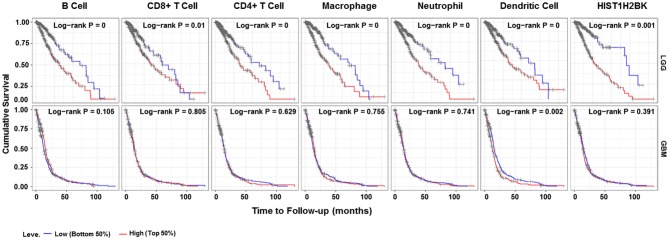
Survival curve for immune cell infiltration. Kaplan–Meier survival curves based on top and bottom sample partitions with 50% immune penetration. Red indicates a high degree of infiltration and blue indicates a low degree of infiltration. *P* < 0.05 was considered significant, and *P* < 0.0001 was reported as 0. GBM, glioblastoma; LGG, low grade glioma.

**Figure 7:**
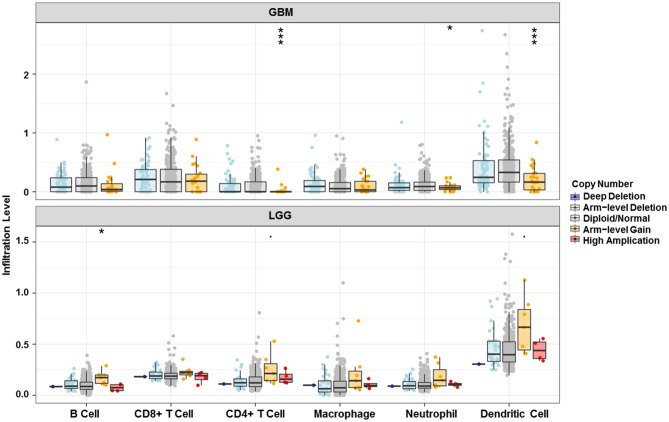
Relationship between copy number variation of HIST1H2BK and immune infiltration level in glioma. *P < 0.05; ***P < 0.001. GBM, glioblastoma; LGG, low grade glioma.

Analysis using TIMER showed that HIST1H2BK was negatively associated with purity and CD8+ T cells, and was positively correlated with dendritic cells in GBM. In low grade glioma (LGG), HIST1H2BK was inversely related to tumor purity, and was positively correlated with B cells, CD4+ T cells, CD8+ T cells, neutrophils, macrophages and dendritic cells (Figure 5 and Table 1). Univariate Cox survival analysis showed that six types of immune cells and HIST1H2BK were indicators of survival of patients with LGG, while only dendritic cells were associated with survival of patients with GBM (Figure 6 and Table 2). Multivariate Cox survival analysis showed that age, macrophages, and HIST1H2BK were independent prognostic indicators for patients with LGG (Table 3), and age and dendritic cells were independent prognostic indicators for patients with GBM (Table 4). Furthermore, only arm-level increases in CNVs of HIST1H2BK were associated with the extent of immune infiltration in glioma immune cells (Figure 7). These results showed that HIST1H2BK was associated with immune infiltration in glioma using external data analysis. These results agreed with those obtained using GSEA analysis from CGGA data. These findings indicated that HIST1H2BK might be a prognostic biomarker of glioma, and may be a target for immunotherapy.

**Table 1:** Association between HIST1H2BK expression and immune infiltration in glioma (partial Spearman correlation).

**Cancer**	**Variable**	**Partial.cor**	***P***
LGG	Dendritic Cell	0.504	**0**
LGG	CD4+ T Cell	0.489	**0**
LGG	Neutrophil	0.471	**0**
LGG	Macrophage	0.422	**0**
LGG	B Cell	0.418	**0**
LGG	Purity	−0.203	**0**
LGG	CD8+ T Cell	0.135	**0.003**
GBM	Dendritic Cell	0.257	**0**
GBM	CD8+ T Cell	−0.249	**0**
GBM	Purity	−0.117	**0.017**
GBM	Macrophage	0.064	0.191
GBM	B Cell	−0.051	0.298
GBM	Neutrophil	0.051	0.301
GBM	CD4+ T Cell	0.03	0.547

*LGG (n = 458), low grade glioma; GBM (n = 402), glioblastoma*.

**Table 2:** Univariate analysis of the correlation among HIST1H2BK expression, immune infiltration, and OS in patients with glioma.

**Cancer**	**Variable**	***P***
LGG	B Cell	**0**
LGG	CD8+ T Cell	**0.01**
LGG	CD4+ T Cell	**0**
LGG	Macrophage	**0**
LGG	Neutrophil	**0**
LGG	Dendritic Cell	**0.001**
LGG	HIST1H2BK	**0**
GBM	B Cell	0.105
GBM	CD8+ T Cell	0.805
GBM	CD4+ T Cell	0.629
GBM	Macrophage	0.755
GBM	Neutrophil	0.741
GBM	Dendritic Cell	**0.002**
GBM	HIST1H2BK	0.391

*LGG (n = 458), low grade glioma; GBM (n = 402), glioblastoma*.

**Table 3:** Multivariate analysis of the correlation of HIST1H2BK expression with immune infiltrates and OS in patients with LGG.

	**Coef**	**HR**	**95% CI_l**	**95% CI_u**	***P*-value**	**Sig**
Age	0.058	1.06	1.042	1.077	0	^***^
Gender (male)	0.162	1.175	0.778	1.775	0.442	
Purity	−0.296	0.744	0.283	1.953	0.548	
B cell	0.201	1.223	0.001	1100.941	0.954	
CD8 T cell	4.192	66.169	0.055	79341.4	0.246	
CD4 T cell	−4.416	0.012	0	55.693	0.305	
Macrophage	5.244	189.42	2.566	13984.56	0.017	^*^
Neutrophil	−6.498	0.002	0	11.785	0.155	
Dendritic Cell	2.27	9.679	0.106	881.085	0.324	
HIST1H2BK	0.677	1.967	1.505	2.572	0	^***^

LGG (n = 458), low grade glioma.

* P < 0.05;

*** *P < 0.001*.

**Table 4:** Multivariate analysis of the correlation of HIST1H2BK expression with immune infiltrates and OS in patients with GBM.

	**Coef**	**HR**	**95% CI_l**	**95% CI_u**	***P*-value**	**Sig**
Age	0.029	1.03	1.021	1.039	0	^***^
Gender (male)	0.071	1.074	0.854	1.351	0.543	
Purity	0.048	1.049	0.539	2.044	0.887	
B cell	−0.499	0.607	0.317	1.161	0.131	
CD8 Tcell	0.357	1.429	0.912	2.241	0.12	
CD4 Tcell	0.181	1.199	0.544	2.639	0.653	
Macrophage	0.422	1.525	0.704	3.3	0.284	
Neutrophil	0.099	1.104	0.442	2.759	0.833	
Dendritic cell	0.552	1.736	1.258	2.396	0.001	^**^
HIST1H2BK	−0.003	0.997	0.9	1.103	0.948	

GBM (n = 402), glioblastoma.

** P < 0.01;

*** *P < 0.001*.

### Co-expression Analysis of HIST1H2BK

**Figure 8:**
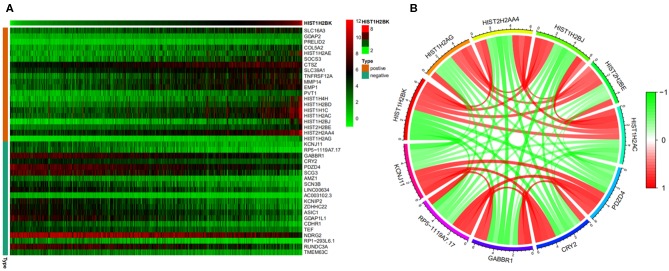
Co-expression analysis of HIST1H2BK using the CGGA database (LGG+GBM, *n* = 749). **(A)** Heatmap of the top 20 genes positively and negatively associated with HIST1H2BK. **(B)** Circular plot of the top five genes positively and negatively related to the HIST1H2BK gene. Green represents negative association, and red represents positive association.

A heatmap (Figure 8A) of the top 20 genes positively and negatively associated with HIST1H2BK was plotted. In addition, a circular plot (Figure 8B) of the top five genes positively and negatively associated with HIST1H2BK was generated. The results showed that HIST1H2BK was positively associated with HIST1H2AG, HIST2H2AA4, HIST1H2BJ, HIST2H2BE, and HIST1H2AC, and was negatively associated with PDZD4, CRY2, GABBR1, rp5-1119a7.17, and KCNJ11 (Supplementary Table 5).

## Discussion

Glioma is the most common primary intracranial neoplasm and is associated with a significant mortality rate (20). Glioblastoma is the most malignant type of glioma due to drug resistance (21). Surgery, radiotherapy, and chemotherapy are the current gold standards for treatment of patients with high-grade glioma (22). Despite aggressive surgery, radiotherapy, and chemotherapy, the average life expectancy of patients with GBM is 12–18 months, with <10% surviving for 5 years (23, 24). Therefore, it is of critical importance to identify biomarkers that could improve prognosis of glioma.

In this study, sequential data filtering was performed from the CGGA database (including LGG and GBM), which resulted in identification of the key gene HIST1H2BK. Then, HIST1H2BK was analyzed using K-M survival analysis, univariate Cox analysis, multivariate Cox analysis, and ROC curve analysis. The relationship between HIST1H2BK expression and clinical characteristics was evaluated. In addition, GSEA and immune infiltration analyses were performed to evaluate the pathways through which HIST1H2BK may be associated with glioma. Finally, co-expression analysis was performed.

Survival analysis filtering, independent prognostic analysis, ROC curve filtering, and clinical relevance filtering were used to screen the key gene HIST1H2BK, which is a member of the HIST1H family of genes. No previous studies have reported a link between HIST1H2BK and glioma or other cancers. However, the HIST1H family of genes has been reported to be associated with cancer. For example, HIST1H2BD, HIST1H2BJ, and HIST1H2BH have been shown to be prognostic indicators for patients with cervical cancer (25).

Kaplan-Meier survival analysis showed that low HIST1H2BK expression was associated with better prognosis in patients with glioma. We showed that HIST1H2BK was a high-risk factor, and could be an independent prognostic indicator in patients with glioma using comprehensive univariate and multivariate Cox analyses. Moreover, ROC curve analysis showed that the AUC values for HIST1H2BK at 1, 3, and 5 years were all >0.7, which indicated that HIST1H2B was a predictor of survival. We have further explored HIST1H2BK as a prognostic biomarker in the TCGA dataset, and no other significant difference was observed in the prognostic analysis for HIST1H2BK in TCGA-GBM. We speculated that the increased expression of HIST1H2BK in all high-grade glioma patients (Figure 3D) reduced the prognosis value of HIST1H2BK, because the expression of histone genes increases sharply during cell replication in high-grade tumors (26, 27). Taken altogether, these results indicated that HIST1H2BK was upregulated in LGG and GBM, and HIST1H2BK had prognostic value in LGG, indicating that HIST1H2BK had important regulatory functions in gliomas.

With The Human Protein Atlas (https://www.proteinatlas.org/), an online tool containing survival data from TCGA and giving users the ability to create publication-quality Kaplan-Meier plots, HIST1H2BK was identified to be a biomarker for renal cancer. Besides, we conducted a literature review of HIST1H2BK, and relative studies showed that the expression level of HIST1H2BK were negative correlated with the prognosis of breast cancer, pancreatic cancer and ovarian cancer (28–30). We speculated that HIST1H2BK may be used as a prognostic indicator for a variety of cancers, and we will explore the protein in a multi-disciplinary way in the future, hoping to find a molecular predictor with great clinical value.

HIST1H2BK participates in the regulation of diverse cellular processes and gene expression through chromatin remodeling, and the overexpression of HIST1H2BK at transcription level would lead to the activation of signaling pathways related to tumor progression (31). For example, LIFR-JAK1-STAT3 signaling pathway would be activated by HIST1H2BK overexpression in breast cancer cells, which leads to aggressiveness in breast cancer (32, 33). Currently, many studies showed that STAT3, as an oncogene, promotes tumor progression in a variety of malignant tumors, including glioma (34, 35). We speculated that the high levels of HIST1H2BK would increase the aggressiveness in glioma via LIFR-JAK1-STAT3 signaling pathway. Besides, Gene set enrichment analysis was performed to obtain further information about the role of HIST1H2BK in tumor progression. The gene ontology terms of HIST1H2BK were generally enriched in B cell related l mediated immunity, humoral immune response and innate immune response. B lymphocyte was recognized to participate in regulating immune response to murine and human tumors (36). Regulatory B cells plays an immunosuppressive role in carcinogenesis and becomes a therapeutic target in solid tumors (37, 38). Recent studies indicated that the B lymphocyte interplays with gliomas and thus influences the prognosis of glioma patients (39). The results of GSEA showed that HIST1H2BK might be involved in tumor progression by regulating the B lymphocyte.

Finally, co-expression analysis showed that HIST1H2BK was positively associated with HIST1H2AG, HIST2H2AA4, HIST1H2BJ, HIST2H2BE, and HIST1H2AC, and was negatively associated with PDZD4, CRY2, GABBR1, RP5-1119A7.17, and KCNJ11. As previously reported, HIST2H2BE may be a promising drug target that could mitigate autoimmune deficiency (40). Moreover, co-immunoprecipitation of HNRNP-K with SERPINA3 correlated with levels of HIST2H2BE transcripts and telomere length in hepatocellular carcinoma (HCC) tissues (41). Yang et al. found that HIST2H2BE was involved in the immune response and cell growth (42). Another study showed that CRY proteins regulated B cell development, and dysregulation of these proteins was associated with autoimmunity (43). These reports suggest that HIST2H2BE and CRY were associated with the immune response. Our study showed HIST1H2BK to be associated with HIST2H2BE and CRY, which indicated that HIST1H2BK might be associated with cellular immunity.

## Conclusion

In conclusion, this study investigated the relationship between HIST1H2BK and glioma prognosis. First, sequential data filtering was used to screen the key gene HIST1H2BK. Then, HIST1H2BK was analyzed for correlations with prognosis and clinical characteristics. The results show that high expression of HIST1H2BK was associated with worse prognosis, and HIST1H2BK was a high-risk factor and could be used as an independent prognostic indicator for patients with glioma. Furthermore, GSEA and analysis of immune infiltration were performed. The results showed that HIST1H2BK could affect the level of immune infiltration in glioma and that upregulation of HIST1H2BK in glioma indicates poor prognosis. Few studies have shown the association between HIST1H2BK and glioma or other cancers, which suggests that this study may provide a scaffold for future development of therapeutic strategies for glioma. However, we did not validate our results using any *in vivo* and *in vitro* models. Future studies should be performed to validate the biological functions and mechanisms of HIST1H2BK.

## Data Availability Statement

The data that support the findings of this work are obtainable from the corresponding author based on reasonable request.

## Author Contributions

WL wrote the manuscript and performed bioinformatics analysis. ZX, JZ, and ZL contributed to manuscript discussion. XG, SF, and SX designed the study, researched the literature, and contributed to figures and tables. WL, YX, and ZL supervised the study and contributed to data analysis. All authors read and approved the final manuscript.

## Conflict of Interest

The authors declare that the research was conducted in the absence of any commercial or financial relationships that could be construed as a potential conflict of interest.
