# Supplementary material for: High Levels of HIST1H2BK in Low-Grade Glioma Predicts Poor Prognosis: A Study Using CGGA and TCGA Data
Source: Front Oncol. 2020 May 8;10:627. doi: 10.3389/fonc.2020.00627 (PMC7225299; doi:10.3389/fonc.2020.00627)
Supplement: Supplementary file 1 [file Data_Sheet_1.PDF]

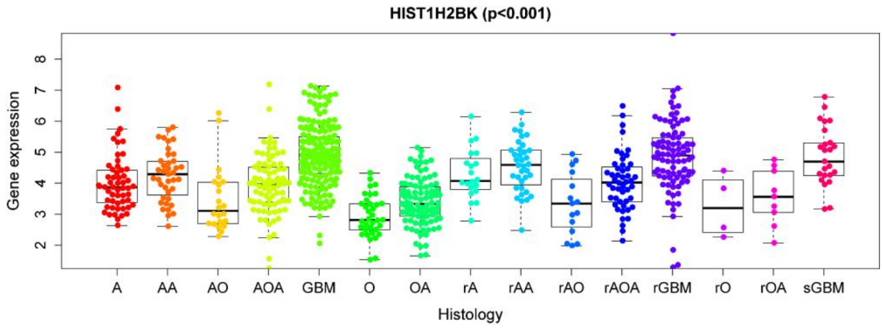

Figure S1. HIST1H2BK was significantly correlated with histology.

Abbreviations:A,low-grade astrocytoma ; AA ,anaplastic astrocytoma; AO,anaplastic oligodendroglioma ; AOA,anaplastic oligoastrocytoma;GBM,glioblastoma; O,oligodendroglioma ;OA,oligoastrocytoma;r,recurrent;s,Secondary .
