# Supplementary material for: High Levels of HIST1H2BK in Low-Grade Glioma Predicts Poor Prognosis: A Study Using CGGA and TCGA Data
Source: Front Oncol. 2020 May 8;10:627. doi: 10.3389/fonc.2020.00627 (PMC7225299; doi:10.3389/fonc.2020.00627)
Supplement: Supplementary file 2 [file Data_Sheet_2.PDF]

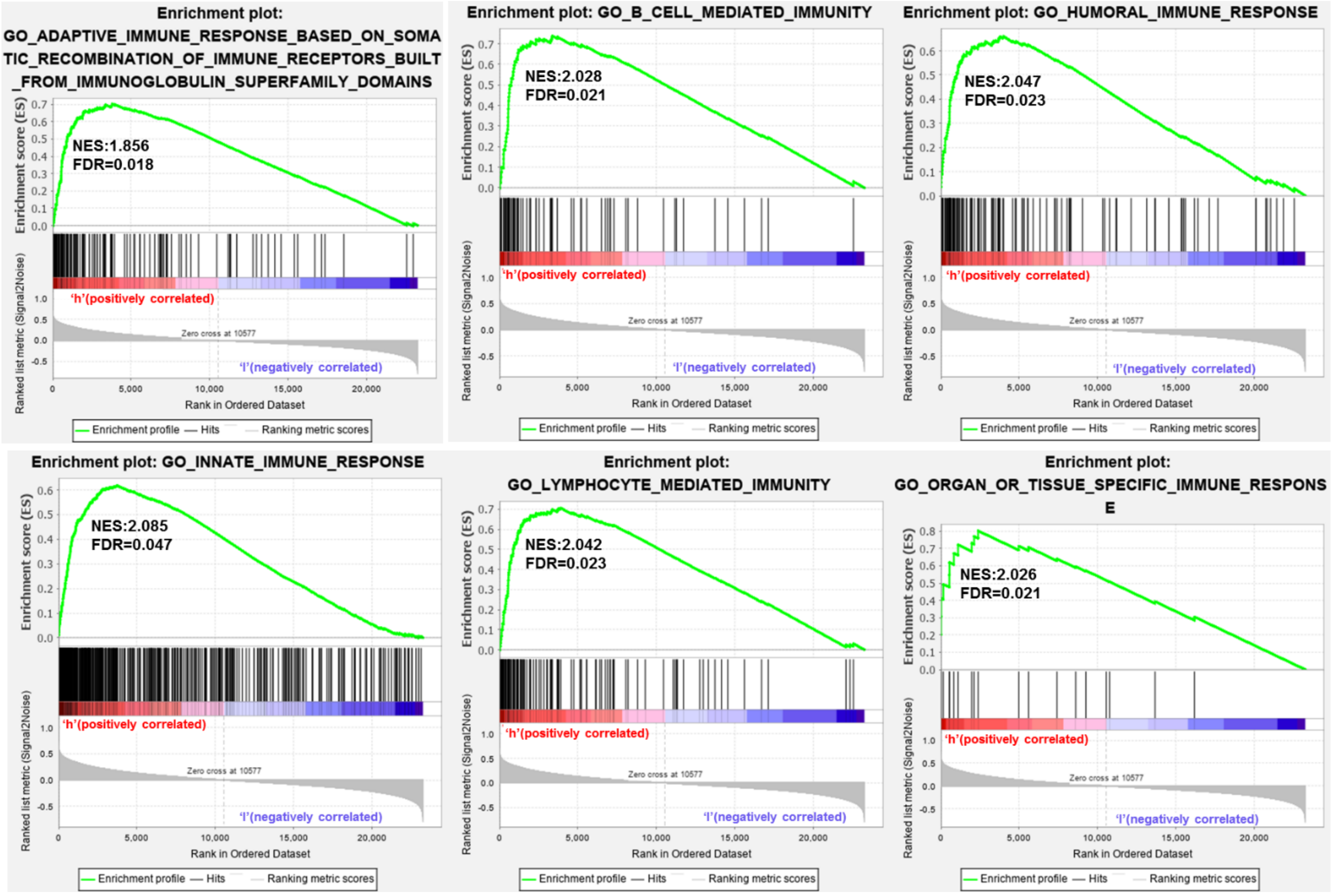

Figure S2. GSEA enrichment analysis (GO). GO adaptive immune response based on somatic recombination of immune receptors built from immunoglobulin superfamily domains, B cell mediated immunity, humoral immune response, innate immune response, lymphocyte mediated immunity GO terms were differentially enriched in HIST1H2BK high expression phenotype; NES, normalized enrichment score; FDR, false discovery rate.
