# Supplementary material for: High Levels of HIST1H2BK in Low-Grade Glioma Predicts Poor Prognosis: A Study Using CGGA and TCGA Data
Source: Front Oncol. 2020 May 8;10:627. doi: 10.3389/fonc.2020.00627 (PMC7225299; doi:10.3389/fonc.2020.00627)
Supplement: Supplementary file 3 [file Data_Sheet_3.PDF]

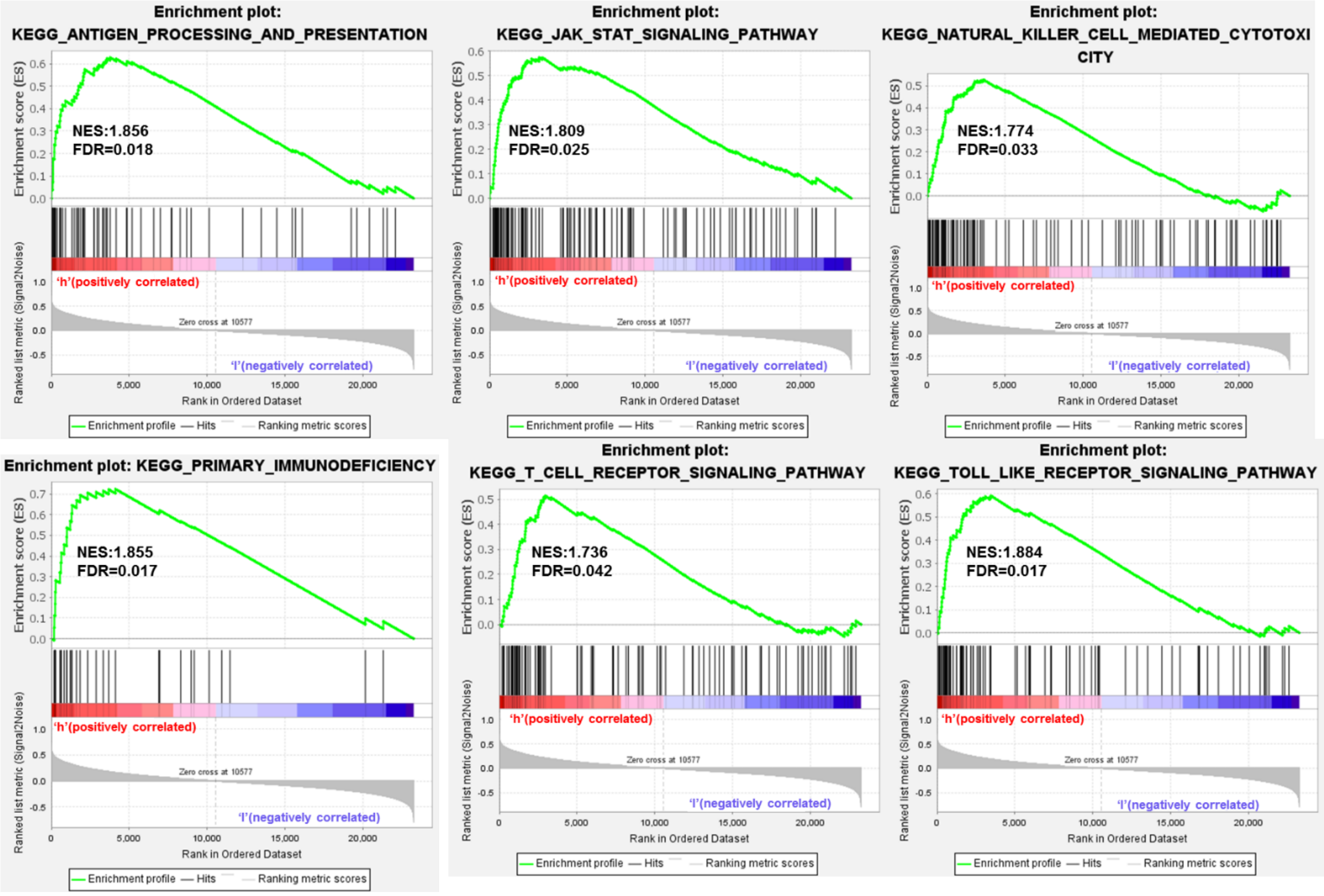


**Figure S3. GSEA enrichment analysis (KEGG).** Antigen processing and presentation, jak stat signaling pathway, natural killer cell mediated cytotoxicity, toll like receptor signaling pathway, primary immunodeficiency pathways were differentially enriched in HIST1H2BK high expression phenotype.

Abbreviations: GSEA, gene set enrichment analysis; NES, normalized enrichment score; FDR, false discovery rate.
