# Supplementary material for: High Levels of HIST1H2BK in Low-Grade Glioma Predicts Poor Prognosis: A Study Using CGGA and TCGA Data
Source: Front Oncol. 2020 May 8;10:627. doi: 10.3389/fonc.2020.00627 (PMC7225299; doi:10.3389/fonc.2020.00627)
Supplement: Supplementary file 4 [file Table_1.DOCX]

**Supplementary Table S1 Survival-related gene filtration in glioma patients.**

| **Gene** | **KM** | **HR** | **HR.95L** | **HR.95H** | **CoxPvalue** |
| --- | --- | --- | --- | --- | --- |
| PLAT | 0 | 1.51 | 1.44 | 1.59 | 1.01E-60 |
| TNFRSF12A | 0 | 1.43 | 1.37 | 1.5 | 4.58E-59 |
| IGFBP2 | 0 | 1.32 | 1.28 | 1.37 | 1.39E-58 |
| KIF2C | 0 | 1.65 | 1.55 | 1.76 | 7.35E-58 |
| EN1 | 0 | 1.62 | 1.53 | 1.72 | 6.85E-57 |
| TAGLN2 | 0 | 1.55 | 1.46 | 1.63 | 2.25E-56 |
| KIF4A | 0 | 1.76 | 1.64 | 1.89 | 7.88E-56 |
| HIST1H2BK | 0 | 1.62 | 1.53 | 1.72 | 1.38E-55 |
| IGF2BP3 | 0 | 1.59 | 1.5 | 1.69 | 1.71E-55 |
| COL5A2 | 0 | 1.49 | 1.42 | 1.56 | 2.54E-55 |
| GAS2L3 | 0 | 1.88 | 1.74 | 2.03 | 5.79E-55 |
| SERPINH1 | 0 | 1.51 | 1.43 | 1.59 | 1.07E-54 |
| GJC1 | 0 | 1.82 | 1.69 | 1.97 | 2.18E-54 |
| SMC4 | 0 | 1.65 | 1.55 | 1.76 | 3.08E-54 |
| COL4A1 | 0 | 1.35 | 1.3 | 1.41 | 3.63E-54 |
| COL4A2 | 0 | 1.38 | 1.32 | 1.44 | 5.51E-54 |
| AURKA | 0 | 1.79 | 1.66 | 1.93 | 2.02E-53 |
| RGS16 | 0 | 1.61 | 1.52 | 1.71 | 2.05E-53 |
| CDCA8 | 0 | 1.71 | 1.6 | 1.83 | 6.51E-53 |
| HOXA5 | 0 | 1.64 | 1.54 | 1.75 | 1.27E-52 |
