# Supplementary material for: High Levels of HIST1H2BK in Low-Grade Glioma Predicts Poor Prognosis: A Study Using CGGA and TCGA Data
Source: Front Oncol. 2020 May 8;10:627. doi: 10.3389/fonc.2020.00627 (PMC7225299; doi:10.3389/fonc.2020.00627)
Supplement: Supplementary file 5 [file Table_2.DOCX]

**Supplementary Table S2 Independent prognosis-related gene filtration in glioma patients.**

| **Id** | **HR** | **HR.95L** | **HR.95H** | **P value** |
| --- | --- | --- | --- | --- |
| CRNDE | 1.27 | 1.19 | 1.35 | 2.82E-14 |
| CNTRL | 1.82 | 1.56 | 2.13 | 5.84E-14 |
| ISL2 | 1.65 | 1.43 | 1.91 | 4.84E-12 |
| PBX3 | 1.48 | 1.32 | 1.66 | 9.69E-12 |
| WDR77 | 2.11 | 1.7 | 2.62 | 1.26E-11 |
| RP4-730K3.3 | 1.84 | 1.54 | 2.2 | 1.76E-11 |
| HIST2H2BE | 1.44 | 1.29 | 1.61 | 3.87E-11 |
| ZCCHC11 | 1.51 | 1.33 | 1.7 | 3.95E-11 |
| GNG12 | 1.38 | 1.25 | 1.51 | 5.50E-11 |
| PHACTR4 | 1.77 | 1.49 | 2.11 | 5.93E-11 |
| TNFRSF19 | 1.32 | 1.22 | 1.44 | 8.52E-11 |
| SMC4 | 1.31 | 1.21 | 1.43 | 1.17E-10 |
| FANCC | 1.65 | 1.42 | 1.92 | 1.18E-10 |
| FAM206A | 1.65 | 1.42 | 1.92 | 1.34E-10 |
| GNAI3 | 1.56 | 1.36 | 1.79 | 1.37E-10 |
| NFE2L3 | 1.54 | 1.35 | 1.76 | 1.63E-10 |
| GPX7 | 1.53 | 1.34 | 1.74 | 1.73E-10 |
| IQGAP2 | 1.33 | 1.22 | 1.46 | 1.78E-10 |
| RNF122 | 1.48 | 1.31 | 1.67 | 2.07E-10 |
| FAM133B | 1.45 | 1.29 | 1.62 | 2.75E-10 |
| HIST1H2BK | 1.30 | 1.17 | 1.44 | 8.65E-10 |
