# Supplementary material for: High Levels of HIST1H2BK in Low-Grade Glioma Predicts Poor Prognosis: A Study Using CGGA and TCGA Data
Source: Front Oncol. 2020 May 8;10:627. doi: 10.3389/fonc.2020.00627 (PMC7225299; doi:10.3389/fonc.2020.00627)
Supplement: Supplementary file 6 [file Table_3.DOCX]

**Supplementary Table S3 ROC-related gene filtration in glioma patients.**

| **Gene** | **Roc** |
| --- | --- |
| PLAT | 0.77 |
| IGFBP2 | 0.76 |
| CHI3L1 | 0.76 |
| TAGLN2 | 0.76 |
| METTL7B | 0.76 |
| TNFRSF12A | 0.75 |
| LOXL1 | 0.75 |
| RP11-189B4.6 | 0.74 |
| PVT1 | 0.74 |
| STK40 | 0.74 |
| HOXA4 | 0.74 |
| ANXA1 | 0.74 |
| IQGAP1 | 0.74 |
| HOXA5 | 0.74 |
| VIM | 0.74 |
| BCAT1 | 0.74 |
| SERPINH1 | 0.73 |
| TUBB6 | 0.73 |
| IGF2BP3 | 0.73 |
| DDOST | 0.73 |
| HIST1H2BK | 0.73 |
