# Supplementary material for: High Levels of HIST1H2BK in Low-Grade Glioma Predicts Poor Prognosis: A Study Using CGGA and TCGA Data
Source: Front Oncol. 2020 May 8;10:627. doi: 10.3389/fonc.2020.00627 (PMC7225299; doi:10.3389/fonc.2020.00627)
Supplement: Supplementary file 7 [file Table_4.DOCX]

**Supplementary Table S4 Clinical characteristics related gene filtration in patients with glioma.**

| **ID** | **PRS_type** | **Histology** | **Grade** | **Gender** | **Age** | **Radio_status** | **Chemo_status** | **IDH_mutation_status** | **1p19q_codeletion_status** | **SigNum** |
| --- | --- | --- | --- | --- | --- | --- | --- | --- | --- | --- |
| HIST1H2BK | 0.0000854 | 1.11E-46 | 3.00E-44 | 0.0224786 | 0.0012009 | 0.9827809 | 8.55E-07 | 4.88E-32 | 6.95E-54 | 8 |
| MED8 | 0.0008285 | 1.71E-44 | 6.71E-46 | 0.0199568 | 3.36E-06 | 0.8420741 | 6.27E-08 | 8.20E-39 | 1.59E-60 | 8 |
| ABCC3 | 0.0012839 | 3.25E-43 | 1.01E-44 | 0.1753062 | 1.9E-07 | 0.4547983 | 5.47E-06 | 1.73E-53 | 4.91E-31 | 7 |
| ABRACL | 0.000033 | 9.10E-37 | 3.22E-39 | 0.3528396 | 0.0000283 | 0.3174976 | 2.90E-10 | 4.33E-54 | 8.87E-27 | 7 |
| AK2 | 0.0000825 | 4.02E-51 | 8.39E-53 | 0.1146626 | 0.0000125 | 0.9690111 | 2.61E-08 | 1.78E-29 | 6.73E-50 | 7 |
| ANXA1 | 0.0000113 | 4.54E-53 | 1.62E-51 | 0.1724792 | 1.16E-07 | 0.4481099 | 6.94E-09 | 1.93E-57 | 1.88E-38 | 7 |
| AURKA | 3.1E-07 | 4.21E-48 | 2.22E-54 | 0.5324102 | 0.0076905 | 0.8273528 | 6.74E-10 | 2.04E-21 | 5.45E-14 | 7 |
| BCAT1 | 0.0217459 | 4.00E-40 | 2.76E-46 | 0.412517 | 1.89E-07 | 0.4004532 | 0.0000203 | 1.44E-74 | 1.73E-31 | 7 |
| CCDC109B | 0.0000391 | 1.67E-50 | 6.78E-49 | 0.4080514 | 3.75E-09 | 0.3468214 | 1.01E-07 | 1.61E-68 | 6.14E-36 | 7 |
| CCNB1 | 1.92E-08 | 2.57E-49 | 2.27E-54 | 0.9327133 | 0.0024955 | 0.4269245 | 3.26E-11 | 2.57E-24 | 4.67E-19 | 7 |
| CD276 | 0.0043909 | 7.53E-45 | 3.40E-50 | 0.3123712 | 1.13E-06 | 0.6882668 | 3.82E-06 | 2.40E-34 | 6.93E-26 | 7 |
| CD58 | 0.0081981 | 3.35E-50 | 6.92E-46 | 0.2782347 | 6.3E-07 | 0.1553047 | 1.15E-06 | 1.11E-69 | 3.10E-50 | 7 |
| CDC20 | 0.0000253 | 2.06E-49 | 3.57E-55 | 0.143873 | 0.0015377 | 0.6654995 | 5.1E-08 | 1.46E-24 | 1.09E-28 | 7 |
| CDC6 | 0.0000213 | 9.46E-45 | 2.30E-50 | 0.7311861 | 0.013721 | 0.2798203 | 5.85E-10 | 2.36E-22 | 5.61E-23 | 7 |
| CDCA2 | 0.0000121 | 3.45E-43 | 2.60E-49 | 0.4924372 | 0.0024111 | 0.1543752 | 1.80E-09 | 3.24E-19 | 4.19E-16 | 7 |
| CDCA8 | 0.0000114 | 1.74E-46 | 7.92E-50 | 0.610503 | 0.0469659 | 0.4311657 | 1.13E-10 | 1.19E-19 | 2.47E-30 | 7 |
| CDKN2C | 0.0000062 | 1.60E-34 | 1.44E-40 | 0.8114936 | 0.0435248 | 0.532279 | 1.12E-09 | 1.59E-22 | 1.05E-14 | 7 |
| CENPA | 2.23E-06 | 7.61E-45 | 4.68E-51 | 0.6341003 | 0.0200284 | 0.561452 | 2.42E-10 | 9.52E-18 | 1.21E-17 | 7 |
| CENPN | 1.55E-09 | 1.33E-42 | 9.29E-47 | 0.5568125 | 0.0025821 | 0.4024931 | 1.63E-08 | 1.63E-19 | 2.73E-11 | 7 |
| CEP112 | 0.0378028 | 6.74E-38 | 3.92E-39 | 0.5065708 | 3.56E-10 | 0.4430953 | 4.19E-06 | 2.42E-76 | 7.41E-38 | 7 |
