# Supplementary material for: High Levels of HIST1H2BK in Low-Grade Glioma Predicts Poor Prognosis: A Study Using CGGA and TCGA Data
Source: Front Oncol. 2020 May 8;10:627. doi: 10.3389/fonc.2020.00627 (PMC7225299; doi:10.3389/fonc.2020.00627)
Supplement: Supplementary file 8 [file Table_5.DOCX]

**Supplementary Table S5. Mean expression level of HIST1H2BK in different glioma types(n=749)**

| Histology | Sample size | Mean expression of HIST1H2BK |
| --- | --- | --- |
| GBM | 176 | 4.92 |
| rGBM | 90 | 4.86 |
| sGBM | 25 | 4.73 |
| rAA | 36 | 4.56 |
| rA | 20 | 4.29 |
| AA | 39 | 4.24 |
| rAOA | 48 | 4.04 |
| A | 55 | 4.01 |
| AOA | 80 | 3.94 |
| rOA | 9 | 3.56 |
| AO | 22 | 3.47 |
| OA | 95 | 3.39 |
| rAO | 15 | 3.33 |
| rO | 4 | 3.26 |
| O | 35 | 2.93 |

Abbreviations:A,low-grade astrocytoma ;AA ,anaplastic astrocytoma;

AO,anaplastic oligodendroglioma ;AOA,anaplastic oligoastrocytoma;

GBM,glioblastoma;O,oligodendroglioma ;OA,oligoastrocytoma;r,recurrent;s,Secondary.
